# MEST Regulates the Stemness of Human Periodontal Ligament Stem Cells

**DOI:** 10.1155/2020/9672673

**Published:** 2020-07-08

**Authors:** Daigaku Hasegawa, Kana Hasegawa, Hiroshi Kaneko, Shinichiro Yoshida, Hiromi Mitarai, Mai Arima, Atsushi Tomokiyo, Sayuri Hamano, Hideki Sugii, Naohisa Wada, Tamotsu Kiyoshima, Hidefumi Maeda

**Affiliations:** ^1^Department of Endodontology, Kyushu University Hospital, Kyushu University, 3-1-1 Maidashi, Higashi-ku, Fukuoka 812-8582, Japan; ^2^Department of Oral Pathology, Faculty of Dental Science, Kyushu University, 3-1-1 Maidashi, Higashi-ku, Fukuoka 812-8582, Japan; ^3^Department of Endodontology and Operative Dentistry, Faculty of Dental Science, Kyushu University, 3-1-1 Maidashi, Higashi-ku, Fukuoka 812-8582, Japan; ^4^Division of General Dentistry, Kyushu University Hospital, Kyushu University, 3-1-1 Maidashi, Higashi-ku, Fukuoka 812-8582, Japan; ^5^OBT Research Center, Faculty of Dental Science, Kyushu University, 3-1-1 Maidashi, Higashi-ku, Fukuoka 812-8582, Japan

## Abstract

Periodontal ligament (PDL) stem cells (PDLSCs) have been reported as a useful cell source for periodontal tissue regeneration. However, one of the issues is the difficulty of obtaining a sufficient number of PDLSCs for clinical application because very few PDLSCs can be isolated from PDL tissue of donors. Therefore, we aimed to identify a specific factor that converts human PDL cells into stem-like cells. In this study, microarray analysis comparing the gene profiles of human PDLSC lines (2-14 and 2-23) with those of a cell line with a low differentiation potential (2-52) identified the imprinted gene mesoderm-specific transcript (MEST). MEST was expressed in the cytoplasm of 2-23 cells. Knockdown of MEST by siRNA in 2-23 cells inhibited the expression of stem cell markers, such as CD105, CD146, p75NTR, N-cadherin, and NANOG; the proliferative potential; and multidifferentiation capacity for osteoblasts, adipocytes, and chondrocytes. On the other hand, overexpression of MEST in 2-52 cells enhanced the expression of stem cell markers and PDL-related markers and the multidifferentiation capacity. In addition, MEST-overexpressing 2-52 cells exhibited a change in morphology from a spindle shape to a stem cell-like round shape that was similar to 2-14 and 2-23 cell morphologies. These results suggest that MEST plays a critical role in the maintenance of stemness in PDLSCs and converts PDL cells into PDLSC-like cells. Therefore, this study indicates that MEST may be a therapeutic factor for periodontal tissue regeneration by inducing PDLSCs.

## 1. Introduction

The periodontal ligament (PDL) is a fiber-rich connective tissue located between the alveolar bone and cementum covering the tooth root, which plays important roles in tooth support as well as nutrition, protection from bacterial attack, sensory input for mastication, and homeostasis [1–4]. However, in most cases, severe damage to PDL tissue caused by deep caries, periodontitis, or trauma results in tooth loss because the current therapies have limited effects and it is difficult to regain complete regeneration [5].

Previous reports have indicated that human PDL tissue contains somatic stem cells [6]. These cells termed as PDL stem cells (PDLSCs) express not only mesenchymal stem cell (MSC) surface markers, such as CD105 and CD146 [6–10], but also various stem cell-related markers, such as p75NTR (the neural crest marker) [10, 11], N-cadherin (the mesenchymal stem cell marker) [10], and NANOG (the embryonic stem cell marker) [11, 12] and possess self-renewal properties [7, 13]. PDLSCs also display a multidifferentiation capacity for osteoblasts, adipocytes, and chondrocytes in vitro similarly to MSCs [6, 14] and possess the capacity to generate cementum- and PDL-like tissues in vivo [6]. Other studies have reported that transplantation of autologous PDLSCs into human and swine periodontal defects regenerates PDL tissue [15, 16]. Thus, it has been considered that the use of PDLSCs in tissue engineering techniques may be a critical method for regenerative periodontal therapy. However, because the percentage of resident stem cells in PDL tissue is very low [17] and isolation of PDLSCs involves tooth extraction, it has been difficult to stably obtain sufficient PDLSCs for research and clinical applications.

Therefore, we considered that a method to address these issues is induction of stem cell populations from PDL cells. Previously, we showed that semaphorin 3A (Sema3A) induces MSC-like properties in human PDL cells [18]. Sema3A-overexpressing PDL cells exhibit an enhanced capacity to differentiate into both osteoblasts and adipocytes, but not chondrocytes, although not having increased expression of all MSC markers. Thus, we attempted to identify a factor in PDLSCs to induce MSC-like properties more effectively.

In this study, we aimed to identify such a factor by microarray analysis to compare gene profiles among three clonal cell lines with different properties. Among them, 2-14 and 2-23 cells strongly express MSC surface markers, such as CD105 and CD146, and possess multidifferentiation capacities for osteoblasts, adipocytes, and chondrocytes in vitro [9–11]. Conversely, another cell line, 2-52, expresses MSC surface markers less than 2-14 and 2-23 cells and exhibits a limited differentiation capacity [18]. We aimed to identify the factor that was more highly expressed in 2-14 and 2-23 cells than in 2-52 cells and examine whether this factor enables conversion of human PDL cells into stem-like cells.

## 2. Materials and Methods

### 2.1. Cell Culture

Clonal cell lines 2-14, 2-23, and 2-52 were obtained from a limiting dilution of a heterogeneous immortalized human PDL fibroblast line. The heterogeneous immortalized human PDL fibroblast line was generated by transduction with both simian virus 40 large T-antigen and human telomerase reverse transcriptase into a human PDL cell population which was isolated from the healthy premolars of a 21-year-old female who visited Kyushu University Hospital for extraction [19]. Cell line 1-17 was also an immortalized human PDL cell line that we established previously [20]. 2-14, 2-23, and 1-17 cells were reported as human PDLSC-like cells in our previous studies [9, 10, 20]. Cells maintained in alpha-minimum essential medium (*α*-MEM; Gibco-BRL, Grand Island, NY) containing 10% fetal bovine serum (FBS; Biowest, Nuaillé, France) (10% FBS/*α*-MEM) at 37°C in a humidified atmosphere with 5% CO_2_ were used in all experiments. All procedures were performed in compliance with the Research Ethics Committee of the Faculty of Dentistry at Kyushu University.

### 2.2. Osteoblastic Differentiation Assay

Cells were seeded at 2 × 10^4^ cells per well in 24-well plates (Becton Dickinson Labware, Lincoln Park, NJ) in 10% FBS/*α*-MEM as control medium (CM) or CM containing 2 mM *β*-glycerophosphate (Sigma, St. Louis, MO), 50 mg/ml ascorbic acid (Nacalai Tesque, Kyoto, Japan), and 1 × 10^−7^ M dexamethasone (Merck Millipore, Darmstadt, Germany) as osteoblastic differentiation medium (DM). Half of the medium was exchanged every 2 or 3 days. After 3 weeks of culture, the cells were fixed with 4% paraformaldehyde (PFA; Merck Millipore) and then washed with distilled water and stained with Alizarin red S as described previously [9]. The area of each Alizarin red S-positive region was imaged under a Biozero digital microscope (Keyence, Osaka, Japan). Total RNA was isolated from each culture after 3 days.

### 2.3. Adipogenic Differentiation Assay

Cells seeded at 2 × 10^4^ cells per well in 24-well plates (Becton Dickinson Labware) were cultivated in 10% FBS/*α*-MEM in the presence of 0.5 mM methylisobutylxanthine (Sigma), 0.5 *μ*M hydrocortisone (Sigma), 60 *μ*M indomethacin (Sigma), and 100 *μ*M ascorbic acid (Nacalai) for 3 weeks as described previously [9]. Lipid-containing fat cells were identified by Oil Red O staining. Total RNA was isolated from each culture after 3 days.

### 2.4. Chondrogenic Differentiation Assay

Cell suspensions of 2.5 × 10^5^ cells per 15 ml polypropylene tube (Thermo Fisher Scientific, Waltham, MA) were centrifuged at 150 × *g* for 5 min, and the cell pellets were cultivated in complete chondrogenic medium with 10 ng/ml recombinant TGF-*β*3 (R&D Systems, Minneapolis, MN) for 4 weeks as described previously [9]. After fixing with 4% PFA in phosphate-buffered saline (PBS), the cell pellets were embedded in paraffin and sectioned into 5 *μ*m thick slices. Alcian blue staining was performed to identify a cartilaginous matrix. Total RNA was isolated from each culture after 3 days.

### 2.5. Flow Cytometric Analysis

The expression of cell surface antigens on 2-14, 2-23, and 2-52 cells was analyzed by flow cytometry. Cells (2 × 10^5^/tube) prepared as a single cell suspension by trypsin/EDTA digestion and resuspended in flow cytometry buffer (R&D Systems) were incubated with antibodies (10 mg/ml) specific for surface markers or isotype control antibodies (10 mg/ml) on ice for 45 minutes. Anti-CD105-PE and anti-CD146-PE antibodies (eBioscience, San Diego, CA) and mouse IgG-PE isotype control were used. The cells were washed with flow cytometry staining buffer and analyzed using an EC800 cell analyzer (Sony Biotechnology, Tokyo, Japan).

### 2.6. Semiquantitative RT-PCR

PCR was performed using Platinum Taq DNA polymerase (Invitrogen, Carlsbad, CA) in a PCR Thermal Cycler Dice (Takara Bio Inc., Shiga, Japan) under the following conditions: 94°C for 2 min and then the appropriate number of cycles at 94°C for 30 s, appropriate annealing temperature for 30 s, 72°C for 30 s, and finally 72°C for 7 min. Primer sequences, annealing temperatures, cycle number, and product sizes for *Type-1 collagen* (*COL-1*), *Periodontal ligament-associated protein-1* (*PLAP-1*), *Periostin* (*POSTN*), and glyceraldehyde3-phosphate dehydrogenase (*GAPDH*) are shown in Table 1. *GAPDH* primers were used as internal standards. All PCR assays were performed within the exponential amplification range. PCR products were separated by electrophoresis on 2% agarose gels (Seakem ME; BioWhittaker Molecular Applications, Rockland, ME) and photographed under ultraviolet excitation after ethidium bromide staining.

### 2.7. Microarray Analysis

Total RNA was isolated from 2-14, 2-23, and 2-52 cells using a TRIzol Reagent (Invitrogen) and purified using the SV Total RNA Isolation System (Promega, Madison, WI), according to the manufacturers' instructions. RNA samples were quantified using a NanoDrop ND-1000 spectrophotometer (Thermo Fisher Scientific). The quality of RNA was checked using an Experion automated electrophoresis station (Bio-Rad Laboratories Inc., Hercules, CA). Microarray analysis using the Agilent Technologies System (Santa Clara, CA) was performed by Cell Innovator, Inc. of the Venture Business Laboratory of Kyushu University (Fukuoka, Japan) as follows. cDNA was amplified and labeled using a Low Input Quick Amp Labeling kit (Agilent Technologies) and hybridized to a SurePrint G3 Human Gene Expression Microarray 8 × 60K v2 (Agilent Technologies). The raw signal intensities of samples (considered as 2-14 vs. 2-52 (compare1) and 2-23 vs. 2-52 (compare2)) were normalized using the quantile algorithm in the preprocess Core library package of the Bioconductor application.

### 2.8. Quantitative RT-PCR

Total cellular RNA was isolated with the TRIzol Reagent. First-strand cDNA was synthesized from 1 *μ*g total RNA using an ExScript RT Reagent kit (Takara). Total RNA was reverse transcribed with random hexamers and ExScript RTase for 15 min at 42°C. The reaction was stopped by incubation for 2 min at 99°C, followed by 5 min at 5°C. PCR was performed using SYBR Green II (Takara) in a Thermal Cycler Dice Real-Time System (Takara) under the following conditions: 95°C for 10 s (initial denaturation) and then 40 cycles at 95°C for 5 s and 60°C for 30 s, followed by a dissociation program at 95°C for 15 s, 60°C for 30 s, and 95°C for 15 s. Primer sequences, annealing temperatures, cycle number, and product sizes for *p75NTR*, *N-cadherin*, *NANOG*, *MEST*, *Cyclin D1* (*CCND1*), *Cyclin E1* (*CCNE1*), *ALP*, *Osterix*, *PPARγ*, *C/EBPα*, *Type-2 collagen* (*COL-2*), *Aggrecan*, *COL-1*, *PLAP-1*, *POSTN*, and *β-actin* are shown in Table 2. *β-Actin* was used as an internal standard. Expression levels of the target genes were calculated using *ΔΔ*Ct values.

### 2.9. Western Blot Analysis

The cells were lysed in buffer containing 50 mM Tris-HCl (pH 6.9; Sigma), 2% sodium dodecylsulfate (SDS; Nacalai), 6% 2-mercaptoethanol (Sigma), and 10% glycerol. Protein samples (20 *μ*g) were subjected to 4%–20% SDS-polyacrylamide gel electrophoresis and subsequently transferred onto an Immuno-Blot PVDF membrane (Bio-Rad). The membrane was treated with a mouse monoclonal anti-*β*-actin antibody (Santa Cruz Biotechnology) at a dilution of 1 : 1000 or goat polyclonal anti-MEST antibody (Abcam) at a dilution of 1 : 200, followed by biotinylated anti-mouse IgG (Nichirei Biosciences, Inc., Tokyo, Japan) or anti-goat IgG (Nichirei) as appropriate. After the membrane was washed thoroughly, the reactive bands were visualized using the ECL Select western blotting detection system (GE healthcare, Buckinghamshire, UK) and Image Quant LAS 500 (GE).

### 2.10. Immunofluorescence Staining

Cells were fixed with 4% PFA and 0.5% dimethyl sulfoxide (Wako) in PBS for 20 min. After blocking with 2% BSA in PBS for 1 h, the cells were incubated for 1 h with a goat polyclonal anti-MEST antibody (Abcam) at a dilution of 1 : 200 or mouse monoclonal anti-Ki67 antibody (Invitrogen) at a dilution of 1 : 100 and then washed with PBS. The cells were then incubated for 1 h with an Alexa Fluor 488-conjugated chicken anti-goat antibody (Invitrogen) at a dilution of 1 : 200. The cells were washed with PBS and then counterstained with DAPI (Vector Laboratories, Burlingame, CA). The cytoskeleton was stained with Phalloidin-Tetramethylrhodamine B isothiocyanate (Sigma). Cells were imaged and analyzed under the Biozero digital microscope.

### 2.11. Small Interfering RNA Transfection

Small interfering RNAs (siRNAs) against MEST (MISSION siRNA) and nontargeting control siRNA (MISSION siRNA Universal Negative Control) were purchased from Sigma. One day before transfection, 2-23 cells were seeded on 24-well plates in Opti-MEM (Invitrogen) containing 10% FBS (10% FBS/Opti-MEM). At 30%-50% confluency, the cells were transfected with siRNA. Twenty picomoles of each siRNA in 50 *μ*l Opti-MEM was mixed with 1 *μ*l preincubated Lipofectamine iMAX (Invitrogen) in another 50 *μ*l Opti-MEM. The mixture was incubated for 20 min at room temperature and then added to the cell culture in 10% FBS/Opti-MEM at 37°C in a humidified atmosphere with 5% CO_2_. After 48 h, the medium was replaced with fresh 10% FBS/*α*-MEM.

### 2.12. WST-1 Proliferation Assay

Cells were seeded in 96-well plates (Becton) at 3 × 10^3^ cells/well in 10% FBS/*α*-MEM and cultured for 24 and 48 h. The cell proliferation rate was measured using the Premix WST-1 Cell Proliferation Assay System (Takara), according to the manufacturer's instructions. Briefly, at the indicated time points, 10 *μ*l WST-1 reagent was added to the culture medium of each well. After 1 h, 100 *μ*l of supernatant was collected from each well, and the absorbance at 450 nm was measured using a microplate reader (ImmunoMini NJ-2300; System Instruments Co., Ltd., Tokyo, Japan).

### 2.13. Gene Transfection and Establishment of Transfected Clones

The coding region of the human MEST cDNA (GenBank accession no. NM_002402.4) was inserted into the pcDNA3.1/Hygro (+) (Invitrogen) expression vector. 2-52 cells were transfected with pcDNA3.1/Hygro (+) alone or with pcDNA3.1/Hygro (+) containing the coding region of the human MEST cDNA using Lipofectamine LTX and Plus Reagent (Invitrogen). These were termed “2-52_empty” and “2-52_MEST,” respectively. 2-52_empty cells were regarded as controls. The candidate clones of 2-52_empty and 2-52_MEST cells were established by selection with hygromycin (Invitrogen). The levels of MEST expression in the clones stably expressing the MEST cDNA were assessed by quantitative RT-PCR and western blot analysis.

### 2.14. Statistical Analysis

All experiments were performed in triplicate or quadruplicate. Values are expressed as the mean ± standard deviation. Statistical analysis was performed using paired Student's *t*-test. A value of *p* < 0.05 was considered as statistically significant.

## 3. Results

### 3.1. Identification of MEST by Microarray Analysis

**Figure 1:**
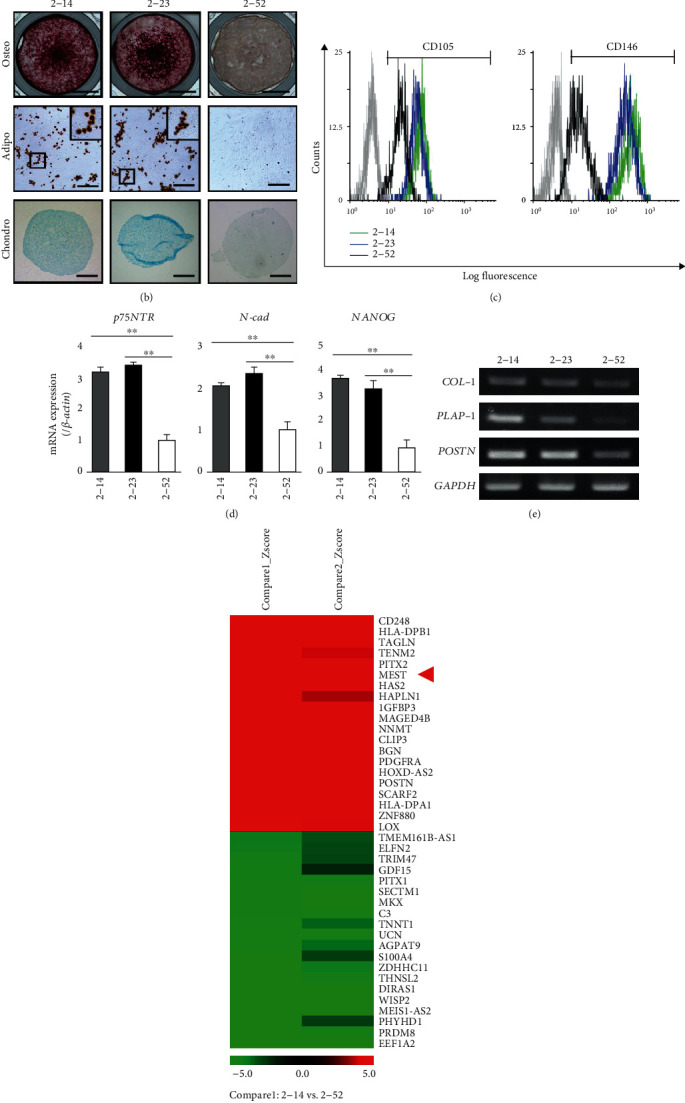
Identification of MEST by microarray analysis. (a–e) The characterization of 2-14, 2-23, and 2-52 cells. (a) Phase-contrast view of three cell lines (bars = 40 *μ*m). (b) The multidifferentiation capacity for osteoblasts, adipocytes, and chondrocytes of three cell lines. Alizarin red S staining of cells cultured in osteoblastic differentiation medium for 3 weeks (top row: bars = 5 mm). Oil Red O staining of cells cultured in adipogenic inductive condition for 3 weeks. Inset: higher magnified view of Oil red O-positive lipid (medium row: bars = 20 *μ*m). Alcian blue staining of cells cultured in chondrogenic inductive condition for 4 weeks (bottom row: bars =200 *μ*m). (c) The expression intensities of CD105 and CD146 of 2-14 (green line), 2-23 (blue line), and 2-52 (black line) cells was demonstrated by flow cytometric analysis. In the gated region, positive cells. (d) The gene expression of *p75NTR*, *N-cadherin*, and *NANOG* in three cell lines was examined by quantitative RT-PCR. It was normalized against *β-actin* expression. All values are means ± S.D. (error bars) of quadruplicate assays. ^∗∗^*p* < 0.01. (e) The gene expression of *Type-1 collagen* (*COL-1*), *PLAP-1*, and *Periostin* (*POSTN*) in three cell lines was examined by semiquantitative RT-PCR. It was normalized against *GAPDH* expression. (f) Microarray analysis comparing gene expression among three cell lines (left column: 2-14 vs. 2-52 (compare1); right column: 2-23 vs. 2-52 (compare2)). Heatmap was generated by MeV software. Rows represent the genes, and columns represent the samples. Colors indicate the distance from the median of each row (red blocks: high; green blocks: low; black blocks: similar expression).

To identify the specific factor for human PDLSCs, we conducted microarray analysis to compare gene expression among three human PDL cell lines, 2-14, 2-23, and 2-52. 2-52 cells exhibited a spindle-like shape, while 2-14 and 2-23 cells exhibited a somewhat rounded shape (Figure 1(a)). 2-14 and 2-23 cells possessed a multidifferentiation capacity for osteoblasts, adipocytes, and chondrocytes, whereas 2-52 cells exhibited a limited differentiation capacity (Figure 1(b)). 2-14 and 2-23 cells highly expressed the MSC surface markers, such as CD105 and CD146 (Figure 1(c) and Supplemental Figure 1a), and stem cell-related markers, such as *p75NTR*, *N-cadherin*, and *NANOG* (Figure 1(d)), whereas 2-52 cells expressed fewer of these stem cell markers than 2-14 and 2-23 cells. In addition, 2-14 and 2-23 cells expressed PDL-related genes, such as *COL-1*, *PLAP-1*, and *POSTN*, and the expression was higher than that of 2-52 cells (Figure 1(e)). From these results, we defined 2-14 and 2-23 cells as human PDLSC lines. Based on the results of microarray analysis (2-14 vs. 2-52 and 2-23 vs. 2-52), we selected the molecules that were highly expressed in both human PDLSC lines and focused on MEST (Figure 1(f)).

### 3.2. Expression of MEST in Human PDLSC Lines

**Figure 2:**
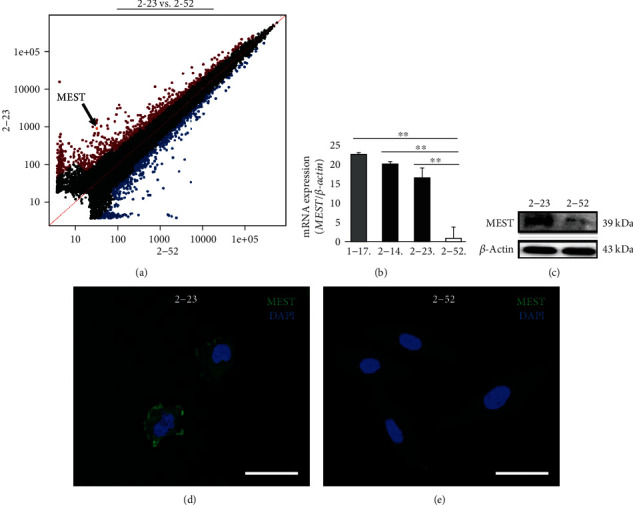
The expression of MEST in human PDLSC lines. (a) Scatterplots representing the expression of genes in 2-23 and 2-52 cells in microarray analysis. (b) The expression of *MEST* mRNA in 1-17 (grey column), 2-14 (left black column), 2-23 (right black column), and 2-52 (white column) cells was assessed by quantitative RT-PCR. It was normalized against *β-actin* expression. All values are means ± S.D. (error bars) of quadruplicate assays. ^∗∗^*p* < 0.01. (c) The expression of MEST protein in 2-23 and 2-52 cells was assessed by western blot analyses. It was normalized against *β*-actin expression. (d, e) The localization of MEST in 2-23 (d) and 2-52 (e) cells was examined by immunofluorescence staining (anti-MEST: green, DAPI: blue; bars: 20 *μ*m).

Microarray analysis showed that the expression level of MEST in 2-23 cells was higher than that in 2-52 cells (Figure 2(a)). Quantitative RT-PCR demonstrated that *MEST* gene was highly expressed in not only 2-14 and 2-23 cells but also 1-17 cells (Figure 2(b)). In addition, western blot analyses revealed that the expression level of MEST protein in 2-23 cells was higher than that in 2-52 cells (Figure 2(c)). Moreover, immunofluorescence staining showed that MEST was expressed in 2-23 cells, and the expression level of MEST in 2-23 cells was higher than that in 2-52 cells (Figures 2(d) and 2(e)). These results indicate that MEST is highly expressed in human PDLSCs.

### 3.3. MEST Knockdown Suppresses the Expression of Stem Cell Markers and the Proliferative Potential in 2-23 Cells

**Figure 3:**
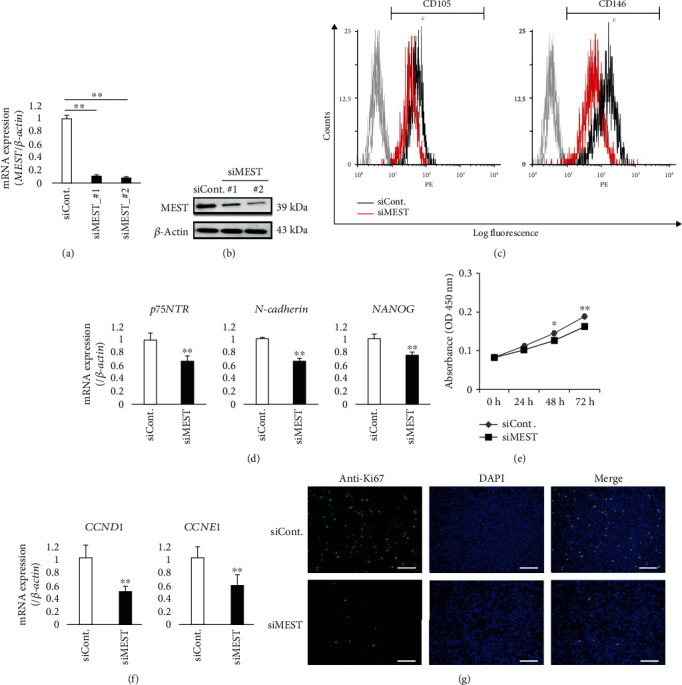
The effects of MEST knockdown on the expression of stem cell markers and the proliferative potential in 2-23 cells. (a, b) The expression of MEST mRNA (a) and protein (b) in 2-23 cells transfected with negative control siRNA (siCont) or MEST siRNA (siMEST_#1 and siMEST_#2) was assessed by quantitative RT-PCR and western blot analyses, respectively. (c) The expression intensities of CD105 and CD146 in 2-23 cells transfected with siCont. (black line) or siMEST_#1 (red line) were demonstrated by flow cytometric analysis. In the gated region, positive cells. (d) The gene expression of *p75NTR*, *N-cadherin*, and *NANOG* in 2-23 cells transfected with siCont. or siMEST was assessed by quantitative RT-PCR. (e) The proliferation of 2-23 cells transfected with negative control siRNA (siCont) or MEST siRNA (siMEST) was examined by WST-1 proliferation assay. The graph shows the time-course of the increase in cell numbers of 2-23 cells transfected with siCont. (grey line) or siMEST (black line). All values are means ± S.D. (error bars) of quadruplicate assays. ^∗∗^*p* < 0.01, ^∗^*p* < 0.05. (f) The gene expression of *Cyclin D1* (*CCND1*) and *Cyclin E1* (*CCNE1*) in 2-23 cells transfected with siCont. or siMEST was assessed by quantitative RT-PCR. (f) The expression of Ki-67 in 2-23 cells transfected with siCont. (upper) or siMEST (lower) was observed by immunocytofluorescence staining (anti-Ki-67: green, DAPI: blue; bars: 500 *μ*m). (a, d, f) In quantitative RT-PCR, the expression levels of these genes were normalized against *β-actin* expression. All values are means ± S.D. (error bars) of quadruplicate assays. ^∗∗^*p* < 0.01, ^∗^*p* < 0.05.

To evaluate the roles of MEST in human PDLSCs, we examined the effects of MEST knockdown by siRNA on stem cell-like properties, such as the expression of stem cell markers, the proliferative potential, and the multidifferentiation capacity. Quantitative RT-PCR and western blot analyses confirmed that the expression of MEST mRNA (Figure 3(a)) and protein (Figure 3(b)) in 2-23 cells was downregulated by 48 h of treatment with MEST siRNA. Flow cytometric analysis demonstrated that the expression of MSC surface markers, such as CD105 and CD146, was lower in 2-23 cells transfected with MEST siRNA (2-23_siMEST) compared with negative control siRNA (2-23_siCont) (Figure 3(c) and Supplemental Figure 1b). In addition, the expression of stem cell-related markers, such as *p75NTR*, *N-cadherin*, and *NANOG*, in 2-23 cells was also downregulated by MEST knockdown (Figure 3(d)). The effect of MEST knockdown on the proliferative potential of 2-23 cells was examined using the WST-1 proliferation assay. We found that MEST knockdown significantly decreased the proliferation rate of 2-23 cells (Figure 3(e)). In addition, we examined the gene expression of cell cycle-related markers *CCND1* and *CCNE1*. Compared with 2-23_siCont cells, the expression level of both genes was downregulated in 2-23_siMEST cells (Figure 3(f)). Moreover, in contrast to 2-23_siMEST cells, immunofluorescence staining showed that 2-23_siCont cells were positive for Ki-67, a typical marker of proliferation (Figure 3(g)). These results suggest that MEST knockdown suppresses the proliferative potential of human PDLSCs.

### 3.4. MEST Knockdown Suppresses the Multidifferentiation Capacity of 2-23 Cells

**Figure 4:**
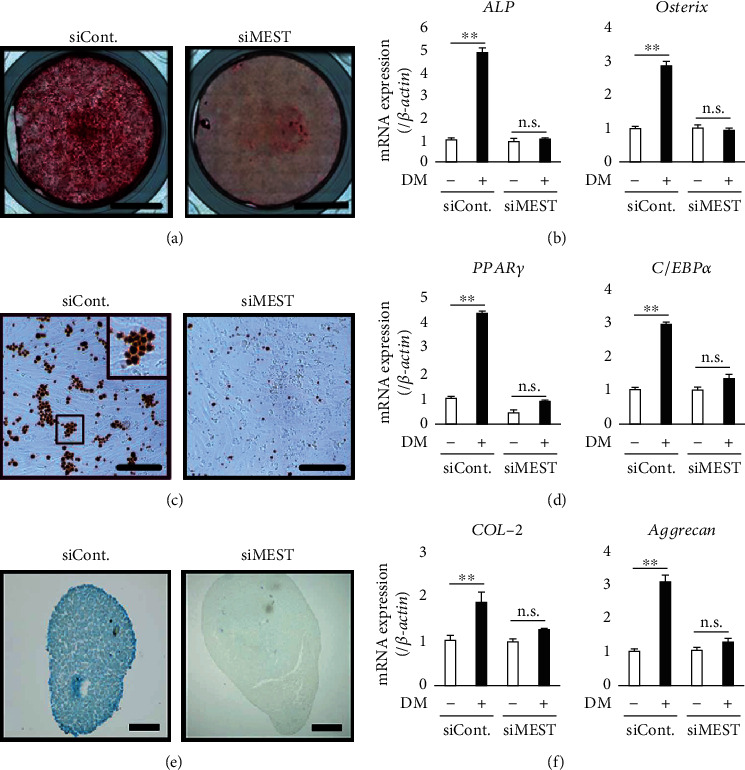
The effects of MEST knockdown on the multidifferentiation capacity in 2-23 cells. (a) The formation of mineralized nodules in 2-23 cells transfected with control siRNA (siCont.) or MEST siRNA (siMEST) was examined by Alizarin red S staining after culture in osteoblastic differentiation medium (DM) for 3 weeks (bars: 5 mm). (b) The gene expression of *ALP* and *Osterix* in 2-23 cells transfected with siCont. or siMEST, which were cultured with or without DM for 3 days, was assessed by quantitative RT-PCR. (c) The lipid vacuoles in 2-23 cells transfected with siCont. or siMEST were examined by Oil red O staining after culture in adipogenic DM for 3 weeks (bars: 20 *μ*m). (d) The gene expression of *PPARγ* and *C/EBPα* in 2-23 cells transfected with siCont. or siMEST, which were cultured with or without DM for 3 days, was assessed by quantitative RT-PCR. (e) The cartilaginous matrix in 2-23 cells transfected with siCont. or siMEST was examined by Alcian blue staining after culture in chondrogenic DM for 4 weeks (bars: 200 *μ*m). (f) The gene expression of *Type-2 collagen* (*COL-2*) and *Aggrecan* in 2-23 cells transfected with siCont. or siMEST, which were cultured with or without DM for 3 days, was assessed by quantitative RT-PCR. (b, d, f) In quantitative RT-PCR, the expression levels of these genes were normalized against *β-actin* expression. All values are means ± S.D. (error bars) of quadruplicate assays. ^∗∗^*p* < 0.01. n.s.: not significant.

We next examined the effect of MEST knockdown on the multidifferentiation capacity of 2-23 cells. The Alizarin red S-positive area of 2-23_siMEST cells cultured in osteoblastic differentiation medium was smaller than that of 2-23_siCont cells (Figure 4(a)). In addition, the expression levels of bone-related genes, such as *ALP* and *Osterix* (Figure 4(b)), were significantly lower in 2-23_siMEST cells cultured in osteoblastic differentiation medium than in 2-23_siCont cells. Similarly, the Oil red O-positive area of 2-23_siMEST cells in adipogenic differentiation medium was smaller than that of 2-23_siCont cells (Figure 4(c)), and the expression levels of adipocyte-related genes, such as *PPARγ* and *C/EBPα* (Figure 4(d)), were significantly lower in 2-23_siMEST cells than 2-23_siCont cells. Moreover, the Alcian blue-positive cartilaginous matrix of 2-23_siMEST cells in chondrogenic differentiation medium was smaller than that of 2-23_siCont cells (Figure 4(e)), and the expression levels of chondrocyte-related genes, such as *COL-2* and *Aggrecan* (Figure 4(f)), were significantly lower in 2-23_siMEST cells than 2-23_siCont cells. These results suggest that MEST knockdown suppresses the multidifferentiation capacity of human PDLSCs.

### 3.5. MEST Overexpression Induces PDLSC-Like Properties in 2-52 Cells

**Figure 5:**
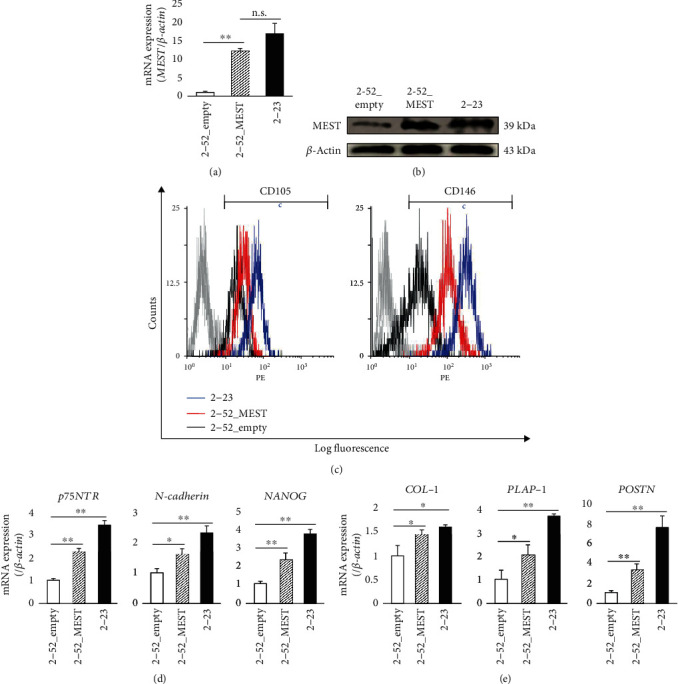
The effects of MEST overexpression on the expression of stem cell markers and PDL-related markers in 2-52 cells. (a, b) The expression of MEST mRNA (a) and protein (b) in 2-52 cells transfected with empty vector (2-52_empty), 2-52 cells transfected with vector coding human MEST (2-52_MEST), and 2-23 cells was assessed by quantitative RT-PCR and western blot analyses, respectively. (c) The expression intensities of CD105 and CD146 in 2-52_empty (black line), 2-52_MEST (red line), and 2-23 cells (blue line) were demonstrated by flow cytometric analysis. In the gated region, positive cells. (d) The gene expression of *p75NTR*, *N-cadherin*, and *NANOG* in 2-52_empty, 2-52_MEST, and 2-23 cells was assessed by quantitative RT-PCR. (e) The gene expression of *COL-1*, *PLAP-1*, and *POSTN* in 2-52_empty, 2-52_MEST, and 2-23 cells was assessed by quantitative RT-PCR. (a, d, e) In quantitative RT-PCR, the expression levels of these genes were normalized against *β-actin* expression. All values are means ± S.D. (error bars) of quadruplicate assays. ^∗∗^*p* < 0.01, ^∗^*p* < 0.05; n.s.: not significant.

**Figure 6:**
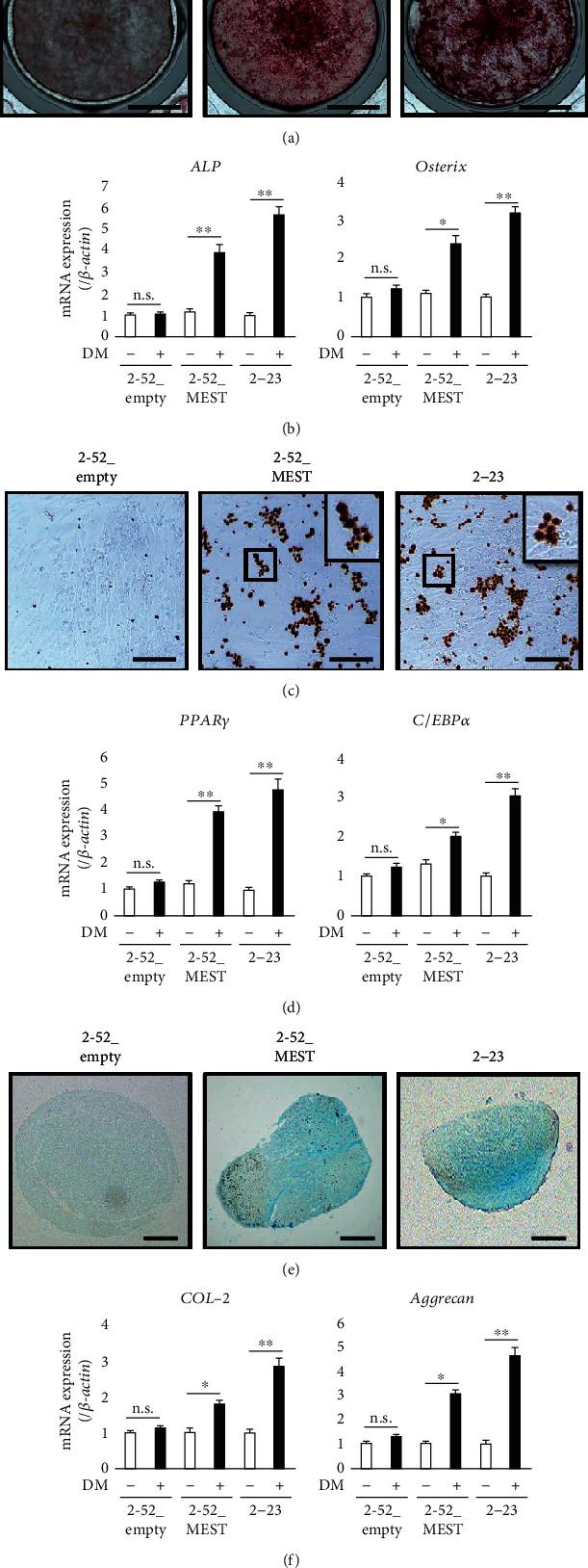
The effects of MEST overexpression on the multidifferentiation capacity in 2-52 cells. (a) The formation of mineralized nodules in 2-52_empty or 2-52_MEST was examined by Alizarin red S staining after culture in osteogenic DM for 3 weeks (bars: 5 mm). (b) The gene expression of *ALP* and *Osterix* in 2-52_empty or 2-52_MEST, which were cultured with or without DM for 3 days, was assessed by quantitative RT-PCR. (c) The lipid vacuoles in 2-52_empty or 2-52_MEST were examined by Oil red O staining after culture in adipogenic DM for 3 weeks (bars: 20 *μ*m). (d) The gene expression of *PPARγ* and *C/EBPα* in 2-52_empty or 2-52_MEST, which were cultured with or without DM for 3 days, was assessed by quantitative RT-PCR. (e) The cartilaginous matrix in 2-52_empty or 2-52_MEST was examined by Alcian blue staining after culture in chondrogenic DM for 4 weeks (bars: 200 *μ*m). (f) The gene expression of *COL-2* (k) and *Aggrecan* (l) in 2-52_empty or 2-52_MEST, which were cultured with or without DM for 3 days, was assessed by quantitative RT-PCR. All values are means ± S.D. (error bars) of quadruplicate assays (*n* = 4, ^∗∗^*p* < 0.01, ^∗^*p* < 0.05. n.s.: not significant).

**Figure 7:**
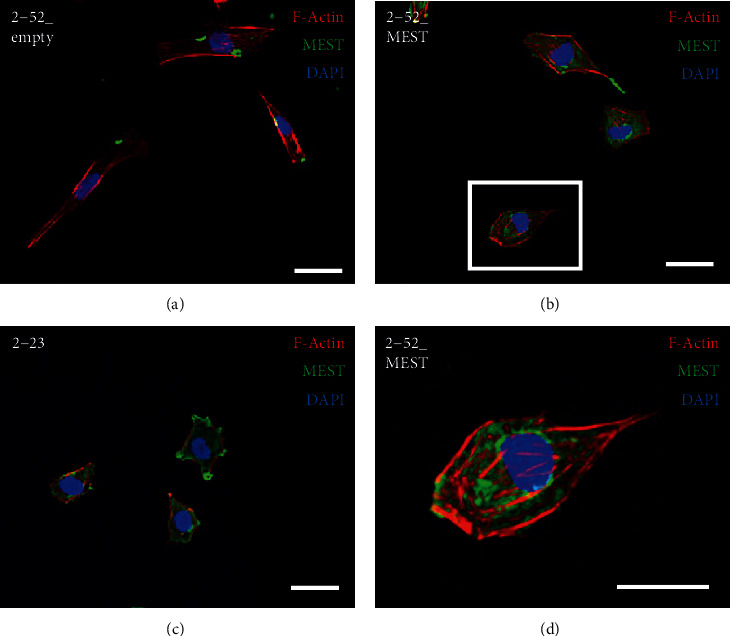
The effects of MEST overexpression on the cell morphology in 2-52 cells. (a–c) The cell morphology was investigated by immunofluorescence staining in 2-52_empty (a), 2-52_MEST (b), and 2-23 cells (c). (d) Highly magnified view of a boxed area in (b). Nuclei were stained with DAPI, and F-actin was stained with phalloidin (F-actin: red; anti-MEST: green; DAPI: blue; bars: 10 *μ*m).

Next, to examine the effects of MEST on stem cell-like properties of human PDL cells, we established 2-52 cells expressing MEST stably, which were transfected with a vector encoding human MEST, termed 2-52_MEST cells. 2-52_MEST cells expressed high levels of the MEST gene and protein compared with 2-52 cells transfected with the empty vector, termed 2-52_empty cells. The expression levels of the MEST gene and protein in 2-52_MEST cells were higher than those in 2-52_empty cells and almost the same as those in 2-23 cells (Figures 5(a) and 5(b)). We found that the expression of MSC surface markers, such as CD105 and CD146 (Figure 5(c) and Supplemental Figure 1c), and stem cell-related markers, such as *p75NTR*, *N-cadherin*, and *NANOG* (Figure 5(d)), was higher in 2-52_MEST cells than in 2-52_empty cells. In addition, the expression of PDL-related genes, such as *COL-1*, *PLAP-1*, and *POSTN*, was also higher in 2-52_MEST cells than in 2-52_empty cells (Figure 5(e)). Moreover, the capacities for osteoblastic, adipogenic, and chondrogenic differentiation in 2-52_MEST cells were higher than those in 2-52_empty cells and almost the same as those in 2-23 cells (Figures 6(a)–6(f)). In addition, interestingly, 2-52_MEST cells exhibited a change in morphology from a spindle shape to a stem cell-like round shape similar to 2-23 cells (Figures 7(a)–7(d)). These results suggested that overexpression of MEST induces PDLSC-like properties in human PDL cells.

## 4. Discussion

In the present study, the loss-of-function analysis using siRNA showed that MEST plays a critical role in the maintenance of stemness in PDLSCs, whereas the gain-of-function analysis employing gene transfer revealed that MEST might convert PDL cells into PDLSC-like cells. This is the first report to demonstrate MEST as a candidate factor that regulates the stemness of human PDLSCs.

First, we identified factors expressed highly in human PDLSC lines by microarray analysis and focused on MEST. MEST is an imprinted gene that plays important roles in embryonic development [21]. Imprinted genes affect all aspects of stem cells from establishment to differentiation [22]. Previous reports have detected imprinted gene expression in adult somatic stem cells and shown that genomic imprinting may be a marker for pluripotency [23, 24]. In our study, MEST was highly expressed in multiple human PDLSC lines including 2-14, 2-23, and 1-17. These findings demonstrated that imprinted genes including MEST might be important in human PDLSCs. Moreover, we found that knockdown of MEST suppressed several stem-like properties in human PDLSCs. This finding confirmed that MEST might play a critical role in the maintenance of stemness in PDLSCs. To date, no factors involved in regulating the stemness of PDLSCs have been reported. Our present study suggested that MEST might be a novel factor that regulates the stemness of PDLSCs. In addition, the findings indicated the possibility that MEST induces stem-like cells.

Previously, transplantation of autologous PDLSCs into human and swine periodontal defects has been performed to regenerate PDL tissue [15, 16]. Furthermore, among other dental stem cells, it has been considered that PDLSCs are the most suitable cells for periodontal tissue regeneration therapy [25, 26]. Therefore, we investigated a method to obtain a sufficient number of PDLSCs for clinical application. Thus far, as a method to acquire PDLSCs, it has been common to separate them from extracted teeth [6]. However, the number of PDLSCs obtained from the extracted teeth of one patient is extremely small [17]. Therefore, as an alternative method, we considered whether PDLSCs could be induced from nonstem cells which are present in the PDL cell population.

Urodele amphibians, such as newts, regenerate amputated limbs and tails by dedifferentiating mature cells into undifferentiated cells [27]. In addition, it has been reported that ceiling cultures induce dedifferentiation of mature adipocytes into MSC-like multipotent cells [28]. Other reports have shown that treatment with reversine induces dedifferentiation of muscle-committed cells into multipotent progenitor cells [29]. However, such findings have not yet been reported for PDL cells. In our recent study, we established a method to dedifferentiate human PDL cells into PDLSC-like cells by transferring a gene that is highly expressed in human PDLSC lines. Previously, we found that Sema3A converts 2-52 cells into MSC-like cells which expressed MSC surface markers and exhibited the differentiation capacity for osteoblasts and adipocytes [18]. However, these cells did not express PDL-related genes. PDL-derived stem-like cells have been reported to express both stem cell-related markers and PDL-related genes [6, 9, 10, 20, 30]. In our present study, we induced PDLSC-like cells that expressed both stem cell-related markers and PDL-related genes by overexpression of the *MEST* gene. To date, there has been no report of PDLSCs expressing both stem cell-related markers and PDL-related genes by transferring a single gene. In addition, these cells exhibited a change in morphology from a spindle shape to a round shape that was similar to human PDLSC lines. These findings indicate that MEST might induce more suitable PDLSC-like cells for periodontal tissue regeneration therapy.

In summary, this study indicates that MEST might enable obtaining sufficient numbers of PDLSCs for clinical application and may be a novel factor with the potential to regenerate PDL tissue efficiently.

## 5. Conclusion

MEST, one of imprinted genes, is highly expressed in human PDLSC lines. Then, MEST knockdown suppresses the expression of stem cell markers, the proliferative potential, and the multidifferentiation capacity for osteoblasts, adipocytes, and chondrocytes in human PDLSC lines. On the other hand, MEST overexpression induces PDLSC-like properties, such as the expression of stem cell markers and PDL-related markers, the multidifferentiation capacity, and the PDLSC-like morphology in human PDL cells. In summary, this study indicates that MEST plays an essential role in the maintenance of stemness in PDLSCs and has the potential to convert PDL cells into stem-like cells. Therefore, MEST may be a novel factor with the potential to regenerate PDL tissue efficiently.

## Figures and Tables

**Table 1:** Primer sequences, annealing temperatures, cycle numbers, and product sizes for semiquantitative RT-PCR.

Gene (abbreviation)	Primer sequence forward/reverse	Annealing temperature (°C)	Semiquantitative RT-PCR cycles	Size of amplified products (bp)
COL-1	5′-CCCGGGTTTCAGAGACAACTTC-3′/5′-TCCACATGCTTTATTCCAGCAATC-3′	61	23	148
PLAP-1	5′-ATGGGAGTCTTGCTAACATACCAC-3′/5′-CAGAAGTCATTTACTCCCACTCTTG-3′	60	30	154
POSTN	5′-CACAACCTGGAGACTGGACA-3′/5′-TGCTTCCTTGTGTGGTCTTT-3′	59	25	198
GAPDH	5′-ACCACAGTCCATGCCATCCAC-3′/5′-TCCACCACCCTGTTGCTGTA-3′	60	20	452

**Table 2:** Primer sequences, annealing temperatures, cycle numbers, and product sizes for quantitative RT-PCR.

Gene (abbreviation)	Primer sequence forward/reverse	Annealing temperature (°C)	Quantitative RT-PCR cycles	Size of amplified products (bp)
p75NTR	5′-CCGAGGCACCACCGACAACC-3′/5′-TGCTTGCAGCTGTTCCACCTCT-3′	60	40	108
N-cadherin	5′-GAAAGACCCATCCACGCCGAGC-3′/5′-TCAGCCGCTTTAAGGCCCTCATT-3′	60	40	101
NANOG	5′-TCCAACATCCTGAACCTCAGCTA-3′/5′-AGGTTCCCAGTCGGGTTCAC-3′	60	40	193
MEST	5′-ATGGGCCATTGGATCCTGTA-3′/5′-CAGAAGGAGTTGATGAAGCCCA-3′	60	40	169
CCND1	5′-AGGAGAACAAACAGATCA-3′/5′-TAGGACAGGAAGTTGTTG-3′	60	40	163
CCNE1	5′-GCCAGCCTTGGGACAATAATG-3′/5′-CTTGCACGTTGAGTTTGGGT-3′	60	40	104
ALP	5′-GGACCATTCCCACGTCTTCAC-3′/5′-CCTTGTAGCCAGGCCCATTG-3′	60	40	137
Osterix	5′-GCCATTCTGGGCTTGGGTATC-3′/5′-GAAGCCGGAGTGCAGGTATCA-3′	60	40	129
PPAR*γ*	5′-TATTCTCAGTGGAGACCGCC-3′/5′-TGAGGACTCAGGGTGGTTCA-3′	60	40	115
C/EBP*α*	5′-GGTGGACAAGAACAGCAAGGA-3′/5′-GTCATTGTCACTGGTCAGCTC-3′	60	40	135
COL-2	5′-CAACCAGGACCAAAGGGACA-3′/5′-ACCTTTGTCACCACGATCCC-3′	60	40	129
Aggrecan	5′-TGGTGATGATCTGGCACGAG-3′/5′-CGTGAGCTCCGCTTCTGTAG-3′	60	40	124
COL-1	5′-CCCGGGTTTCAGAGACAACTTC-3′/5′-TCCACATGCTTTATTCCAGCAATC-3′	60	40	148
PLAP-1	5′-ATGGGAGTCTTGCTAACATACCAC-3′/5′-CAGAAGTCATTTACTCCCACTCTTG-3′	60	40	154
POSTN	5′-CACAACCTGGAGACTGGACA-3′/5′-TGCTTCCTTGTGTGGTCTTT-3′	60	40	198
*β*-Actin	5′-ATTGCCGACAGGATGCAGA-3′/5′-GAGTACTTGCGCTCAGGAGGA-3′	60	40	89

## Data Availability

All data used to support the findings of this study are included within the article.

## Supplementary Materials

Supplementary Materials Supplemental Figure 1: the quantification of mean fluorescence intensities of the MSC surface markers in flow cytometric analysis. (a–c) The quantitative value of mean fluorescence intensities of CD105 and CD146 in 2-14 (grey column), 2-23 (black column), and 2-52 cells (white column) is shown in (a). That in 2-23 cells transfected with siCont. (black column) or siMEST_#1 (black and white striped column) is shown in (b). That in 2-52_empty (white column), 2-52_MEST (black and white striped column), and 2-23 cells (black column) is shown in (c).
