# Supplementary figures and images for: MEST Regulates the Stemness of Human Periodontal Ligament Stem Cells
Source: Stem Cells Int. 2020 Jul 8;2020:9672673. doi: 10.1155/2020/9672673 (PMC7366229; doi:10.1155/2020/9672673)

## SUPPLEMENTAL FIGURE 1

(a)

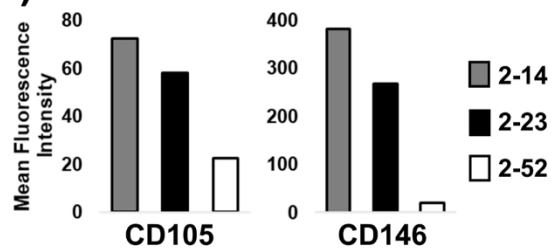

(b)

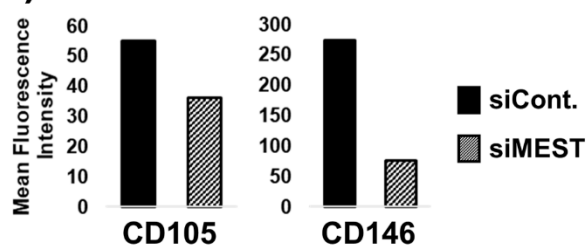

(c)

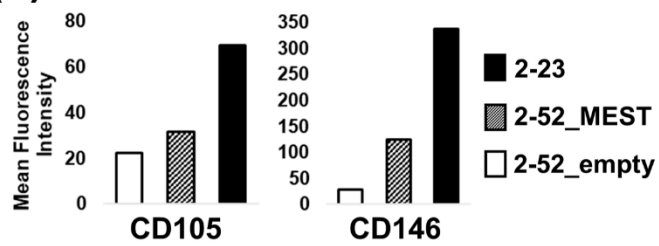

Supplement: Supplementary Materials — Supplemental Figure 1: the quantification of mean fluorescence intensities of the MSC surface markers in flow cytometric analysis. (a–c) The quantitative value of mean fluorescence intensities of CD105 and CD146 in 2-14 (grey column), 2-23 (black column), and 2-52 cells (white column) is shown in (a). That in 2-23 cells transfected with siCont. (black column) or siMEST_#1 (black and white striped column) is shown in (b). That in 2-52_empty (white column), 2-52_MEST (black and white striped column), and 2-23 cells (black column) is shown in (c). [file 9672673.f1.pdf]
